# Cancer-associated fibroblasts promote prostate tumor growth and progression through upregulation of cholesterol and steroid biosynthesis

**DOI:** 10.1186/s12964-019-0505-5

**Published:** 2020-01-24

**Authors:** Hannes Neuwirt, Jan Bouchal, Gvantsa Kharaishvili, Christian Ploner, Karin Jöhrer, Florian Pitterl, Anja Weber, Helmut Klocker, Iris E. Eder

**Affiliations:** 1 Department of Internal Medicine IV - Nephrology and Hypertension, Medical University of Innsbruck, Innsbruck, Austria; 2 Department of Clinical and Molecular Pathology, Institute of Molecular and Translational Medicine, Palacky University and University Hospital, Olomouc, Czech Republic; 3 Department of Plastic, Reconstructive and Aesthetic Surgery, Medical University of Innsbruck, Innsbruck, Austria; 4 Tyrolean Cancer Research Institute, Innsbruck, Austria; 5 Salzburg Cancer Research Institute, Laboratory for Immunological and Molecular Cancer Research, Salzburg, Austria; 6 Institute of Legal Medicine, Medical University of Innsbruck, Innsbruck, Austria; 7 Department of Urology, Division of Experimental Urology, Medical University of Innsbruck, Anichstrasse 35, 6020 Innsbruck, Austria

**Keywords:** Prostate cancer, Castration resistance, Antiandrogens, HMGCS2, AKR1C3, Simvastatin, Cholesterol, Steroid metabolism

## Abstract

**Background:**

Androgen receptor targeted therapies have emerged as an effective tool to manage advanced prostate cancer (PCa). Nevertheless, frequent occurrence of therapy resistance represents a major challenge in the clinical management of patients, also because the molecular mechanisms behind therapy resistance are not yet fully understood. In the present study, we therefore aimed to identify novel targets to intervene with therapy resistance using gene expression analysis of PCa co-culture spheroids where PCa cells are grown in the presence of cancer-associated fibroblasts (CAFs) and which have been previously shown to be a reliable model for antiandrogen resistance.

**Methods:**

Gene expression changes of co-culture spheroids (LNCaP and DuCaP seeded together with CAFs) were identified by Illumina microarray profiling. Real-time PCR, Western blotting, immunohistochemistry and cell viability assays in 2D and 3D culture were performed to validate the expression of selected targets in vitro and in vivo. Cytokine profiling was conducted to analyze CAF-conditioned medium.

**Results:**

Gene expression analysis of co-culture spheroids revealed that CAFs induced a significant upregulation of cholesterol and steroid biosynthesis pathways in PCa cells. Cytokine profiling revealed high amounts of pro-inflammatory, pro-migratory and pro-angiogenic factors in the CAF supernatant. In particular, two genes, 3-hydroxy-3-methylglutaryl-Coenzyme A synthase 2 (HMGCS2) and aldo-keto reductase family 1 member C3 (AKR1C3), were significantly upregulated in PCa cells upon co-culture with CAFs. Both enzymes were also significantly increased in human PCa compared to benign tissue with AKR1C3 expression even being associated with Gleason score and metastatic status. Inhibiting HMGCS2 and AKR1C3 resulted in significant growth retardation of co-culture spheroids as well as of various castration and enzalutamide resistant cell lines in 2D and 3D culture, underscoring their putative role in PCa. Importantly, dual targeting of cholesterol and steroid biosynthesis with simvastatin, a commonly prescribed cholesterol synthesis inhibitor, and an inhibitor against AKR1C3 had the strongest growth inhibitory effect.

**Conclusions:**

From our results we conclude that CAFs induce an upregulation of cholesterol and steroid biosynthesis in PCa cells, driving them into AR targeted therapy resistance. Blocking both pathways with simvastatin and an AKR1C3 inhibitor may therefore be a promising approach to overcome resistances to AR targeted therapies in PCa.

Video abstract

## Background

Prostate cancer (PCa) is one of the four most common types of cancer in Europe in 2018 [1]. Treatment options mainly depend on whether the tumor is localized or metastatic. Localized PCa can be managed by active surveillance, surgical removal of the prostate or radiotherapy. For metastatic PCa, androgen deprivation treatment (ADT) accounts as an important backbone therapy. ADT is based on the blockade of the androgen signaling cascade and in general has a high response rate [1]. Nevertheless, 20–35% of tumors recur as castration-resistant prostate cancer (CRPC) within 5 years [2]. Docetaxel-based chemotherapy has long been the only treatment option to prolong life of patients with CRPC [3]. Nowadays, a panel of new drugs is available as adjuvant therapy even for those patients. Based on the fact that the androgen receptor (AR) is one of the most critical oncogenes in CRPC [4], several AR-targeted therapies including the antiandrogens enzalutamide [5] and abiraterone [6] have emerged. These antiandrogens block the action of androgens or intervene with androgen synthesis to inhibit the activation of the AR. Enzalutamide, for instance, prevents binding of androgens to the AR as well as nuclear translocation and DNA binding of the AR and was shown to increase overall survival of patients who progressed during docetaxel therapy [7, 8]. However, several years of clinical use of these AR targeted therapies showed that resistances also inevitably occur with antiandrogens (reviewed by [9]). Investigating how the tumor cells manage to develop escape mechanisms against these therapies is very important. Resistance to antiandrogens has been previously associated with expression of constitutively active AR variants lacking the ligand-binding domain, overexpression of several other oncogenes like glucocorticoid receptor (GR), NFkB, signal transducer and activator of transcription 3 (STAT3), Snail and Twist, and mutations within the AR gene (AR F876 L), which convert antiandrogens into agonists (reviewed by [10]). Overall, however, the mechanisms underlying antiandrogen resistances are still incompletely understood.

In a previous study we showed that PCa cells become less responsive to enzalutamide when they are co-cultured as tumor spheroids in a 3-dimensional environment together with cancer-associated fibroblasts (CAFs) [11]. In this study, we performed gene expression profiling of these co-culture spheroids and revealed that CAFs induce a significant upregulation of cholesterol metabolism and steroid biosynthesis in PCa cells. In particular, we identified two genes, 3-hydroxy-methyl-glutaryl CoA synthase 2 (HMGCS2) and aldo-ketoreductase 1C3 (AKR1C3), which were significantly upregulated in PCa cells upon co-culture with CAFs and which were also significantly elevated in human PCa specimens compared to benign tissue. Inhibiting these two molecules in various 2D and 3D cell culture models further demonstrated their putative role in the progression of PCa cells to CRPC and antiandrogen resistance. Most notably, dual targeting of cholesterol and steroid biosynthesis with simvastatin, a commonly prescribed cholesterol synthesis inhibitor, and an inhibitor against AKR1C3 had the strongest growth inhibitory effect, suggesting this as a promising strategy to treat CRPC.

## Material and methods

### Cell lines and reagents

LNCaP and CWR22Rv1 were obtained from the American Type Culture Collection (ATCC, Rockville, MD). DuCaP PCa cells were obtained from Prof. J. Schalken (Center for Molecular Life Science, Nijmegen, The Netherlands). These three cell lines were cultured in RPMI 1640 (Lonza) supplemented with 10% fetal calf serum (FCS) (Gibco), 1% penicillin/streptomycin (Lonza), and 1x GlutaMAX™ (Gibco). The androgen-ablated subline LNCaPabl was previously established by long-term culture in androgen-ablated medium [12] and maintained in RPMI 1640 (Lonza) with 10% charcoal-stripped (CS) FCS and 1% penicillin and streptomycin. Immortalized CAFs [13] stably expressing green fluorescent protein (GFP) have been previously established [11] and were grown in DMEM with 10% FCS and 1% penicillin and streptomycin and 1x GlutaMAX™ (Gibco). The enzalutamide resistant (EnzaR) cell lines DuCaP EnzaR and LNCaPabl EnzaR have been previously established by long-term treatment with 8 μM enzalutamide [14]. All cells were cultivated at 37 °C in a humidified atmosphere with 5% CO_2_. Enzalutamide (MedChemExpress), simvastatin (Sigma) and the AKR1C3 inhibitor (3-(4-trifluoromethyl)phenylamino) benzoic acid, Calbiochem) were dissolved in dimethyl sulfoxide (DMSO).

### 3D spheroid growth and viability assay

To obtain 3D spheroids, cells were cultured in 96-well Perfecta 3D hanging drop plates (Sigma) at 7500 cells per drop in 40 μl culture medium as previously described [11]. Co-culture spheroids were produced by seeding prostate tumor cells and CAFs at a ratio of 1:1 as previously optimized [11]. Images were taken with a JuLI Smart Fluorescence Cell Imager microscope (NanoEntek). Culture media were replenished every 96 h. To investigate the influence of CAF-conditioned medium on gene expression, 7500 cells were seeded into 96 well hanging drop plates in 40 μl medium supplemented with 10% FCS. After 72 h, supernatant of wells was pooled (*n* = 48), two parts of supernatant were mixed with one part of fresh medium and added to the tumor cells. To assess 3D spheroid formation and growth assays, cells were cultured in 96 well ULC ultra-low attachment plates (Costar, 7007) where spheroids were monitored automatically using IncuCyte® S3 Live-Cell Analysis System. Cell viability was determined with CellTiterGlo® assay (Promega) according to the manufacturer’s instructions.

### Gene expression analysis and microarray profiling

Cells were seeded into 75cm^2^ cell culture flasks or into 3D 96 well hanging drop plates to form spheroids. Medium was exchanged at days 4 and 6 of culture. After 8 days, cells and spheroids were harvested with trypsin/EDTA, pelleted and shock-frozen in liquid nitrogen. Total RNA was extracted with innuPREP Micro RNA Kit (Analytik Jena, Austria) and RNA quality was verified on an Agilent 2100 bioanalyzer. Hybridization onto Illumina_Human HT-12_v4_r2 microarrays as well as data mining was conducted by Prof. Holger Sültmann (DKFZ, Heidelberg, Germany). Genes with a corrected *P*-value less than 0.05 and a fold change greater than two were considered significantly differentially expressed. Molecular signatures were determined from three biological replicates in a pathway (KEGG pathway annotation) and network context using gene ontology (GOTerm) as provided by the SOURCE tool (https://source-search.princeton.edu/). Datasets are available under digital supplemental data (Additional file 1. Array Data 2D vs 3D.xlsx, Additional file 2 Array Data Cocultures.xlsx). Potential targets were selected on whether they were significantly up- or down-regulated (at least 2-fold) in 3D spheroid versus 2D culture of LNCaP, DuCaP cells and CAFs and in 3D spheroids of tumor cells versus 3D co-culture spheroids where tumor cells were cultured together with CAFs.

### Real time quantitative RT-PCR (qPCR)

Cells and spheroids were harvested as previously described [11]. To validate gene expression in PCa cells after co-culture with CAFs, co-culture spheroids were harvested, pooled (*n* = 48) and digested with trypsin/EDTA. After centrifugation, cells were re-suspended in PBS with 0.1% FCS. GFP-labeled CAFs and tumor cells and then separated by fluorescence-assisted cell sorting on a FACSAria (BD Biosciences) based on GFP expression of CAFs as described previously [11]. Sorted tumor cells were directly harvested in lysis solution (innuPREP DNA/RNA Mini Kit, Analytik Jena, Austria). RNA was quantified with the NanoDrop ND-2000c (Thermo Scientific). Extracted RNA was converted to cDNA by reverse transcription using SuperScript III reverse transcriptase (Invitrogen). We used TaqMan® gene expression assays for quantification of HMGCS2 (Hs00985427_m1), AKR1C3 (Hs00366267_m1) and the endogenous control hydroxymethylbilane synthase (HMBS, Hs00609297_m1). qPCR was carried out with ABI Prism 7500 Fast RT-PCR System (Applied Biosystems) cycler. Fold change in gene expression was determined using the mathematical model ratio 2^-ΔΔCT^ [15]. Values of genes of interest (GOI) were determined relative to HMBS.

### Western blotting

Cells and spheroids were harvested as previously described [11]. Whole cell lysates were generated using Tris Glycine SDS sample buffer (Gradipore) by shaking at room temperature for 1 h and further processed via SDS-PAGE as described previously [16]. The following primary antibodies were used: anti-AKR1C3 (clone NP6.G6.A6, 1:500, Sigma), anti-HMGCS2 (mitochondrial) (ab137043, 1:300, Abcam), and anti-glyceraldehyde-3-phosphate dehydrogenase (GAPDH, 1:50000, Millipore). Visualization and quantification of protein bands were performed with Image Studio software Version 5.2 (LI-COR Biosciences).

### Cytokine profiling

CAFs (8000 cells per well) were seeded into 96 well hanging drop plates in 40 μl cell culture medium per well. After 4 days, supernatant was taken (30 μl per well), pooled (*n* = 48), centrifuged and stored at − 80 °C. Spheroids were harvested, trypsinized and cells were counted. Supernatant was loaded onto RayBio® Human Cytokine Antibody Array G-Series 1000 (RayBiotech, Norcross, GA) which facilitates the detection of 120 targets. The arrays were processed according to the manufacturer’s instructions. Relative fluorescent intensity of spots was scanned with the GenePixx 4000B microarray scanner (Molecular Devices. USA) and specific signal intensities at 532 nm were normalized to background (standard medium). CAF-conditioned media were examined in triplicates from 3 independent experiments. Values are depicted as mean signal intensity with SEM.

### Doxycycline-inducible stable knockdown of HMGCS2

The target sequence of a highly efficient human HMGCS2-specific 29-mer shRNA was reported previously: CGTCTGTTGACTCCAGTGAAGCGCATTCT [17]. Complementary shRNA oligonucleotides directed against human HMGCS2 were cloned into a pENTR-THT vector and sequence-verified THT-shRNA cassettes were recombined into the GATEWAY-based lentiviral doxycycline-regulated conditional RNAi vector pGLTR-X as previously described [18]. To produce lentiviral particles, HEK293T cells were grown in 6-well-plates until 70–80% confluency and transfected with the constructs. The virus particles were harvested 48 h after transfection by collecting the supernatant and filtering it through a 0.2 μm filter. The supernatant was diluted with cell culture medium (1:1) and added to LNCaPabl cells with 1 μg/ml polybrene (Sigma-Aldrich). Successful knockdown of HMGCS2 was confirmed by Western blotting.

### Exogenous overexpression of HMGCS2

HMGCS2 was overexpressed in LNCaP cells, which exhibit low to undetectable expression of HMGCS2 using a pcDNA3-HMGCS2 plasmid (Origene). Cells (500,000 cells/well) were seeded into a 6-well plate and incubated overnight. Transfection was conducted with lipofectamine 3000 (Thermo Fisher Scientific). To assess the effect of HMGCS2 overexpression on 3D spheroid growth, cells were seeded in 96 well ULC ultra-low attachment plates (Corning) at 50 μl per well. Following centrifugation of the plate, the mixture of the HMGCS2 plasmid and lipofectamine 3000 was added at 50 μl/well to the cells. After 4 days of spheroid formation, enzalutamide treatment was started.

### Immunohistochemistry

The study was approved by the Ethical Committee of the University Hospital and Faculty of Medicine and Dentistry, Palacky University in Olomouc (Ref. No. 127/14). Formalin-fixed, paraffin-embedded human prostate tumor samples were obtained after radical prostatectomies between years 1998 and 2011 and archived. Clinicopathological information is given in Table 1. Samples were immunostained with appropriate antibodies according to standard techniques: AKR1C3 (mouse monoclonal, clone NP6.G6.A6, Sigma-Aldrich; microwave antigen retrieval method in citrate buffer, pH 6.0) and HMGCS2 (rabbit monoclonal, clone EPR8642, Abcam; EnVision FLEX target retrieval method in Tris/EDTA, pH 9.0). Target expression was assessed semi-quantitatively by a pathologist using the histoscore method where the percentage of positive cells (0–100%) was multiplied by staining intensity (0–3), which resulted in a final score between 0 and 300 (H-score).

**Table 1:** Clinical and pathologic characteristics of patients

Parameter	Range	Tumor stage (number of patients)			
		pT1–2	pT3–4	N1	Sum
Number of patients		21	26	20	67
Age (years)	49–59	8	8	7	23
	60–69	11	15	12	38
	70–75	2	3	1	6
Tumor grade (Gleason score)	≤ 6	7	3	1	11
	7	13	13	9	35
	≥ 8	1	10	10	21
Serum PSA (ng/mL)	< 4	5	2	0	7
	4–10	12	11	5	28
	> 10	4	13	15	32

### CellTiter Glo proliferation assay

Cells were seeded into 96 well plates (Sarstedt). Cell proliferation was determined by adding 10 μl of CellTiterGlo substrate to each well and measuring relative luminescence units (RLU) with Cytation 5 fluorescent plate reader. Values were corrected with the blank (only medium).

### Statistical analysis

Statistical differences were calculated with Mann Whitney U test using SPSS (V15.0). ANOVA was used to compare more than two groups. Compared groups are given in the figures and/or figure legends and significances are encoded as follows: *p < 0.05; **p < 0.01; ***p < 0.001. Data are presented as mean plus standard error of the mean (SEM) from three independent experiments unless otherwise stated.

## Results

### Prostate cancer cells acquire a typical phenotype in 3D spheroid culture with high expression of genes annotated to cell-to-cell and cell-to-ECM interaction but low expression of cell cycle genes

**Fig. 1:**
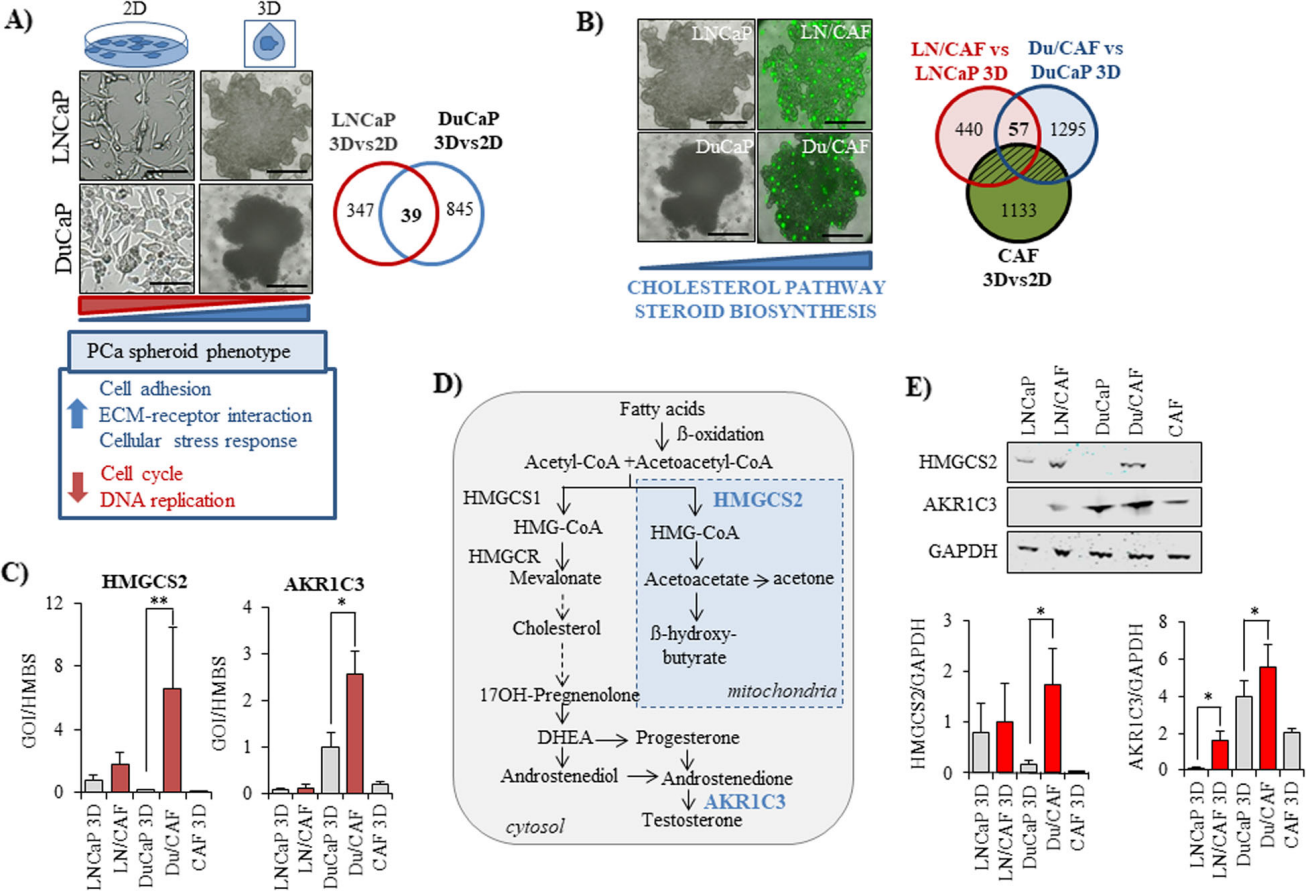
Gene expression patterns of PCa spheroids with and without cancer-associated fibroblasts (CAFs). **a** Representative phase contrast images are shown for LNCaP and DuCaP PCa cells grown either in 75cm^2^ culture flasks (2D) or as 3D spheroids in 96 well hanging drop plates in the absence and (**b**) presence of GFP-expressing CAFs (magnification 10x, scale bar: 500 μm). Alterations in mRNA gene expression were determined via Illumina microarray analysis and the number of differentially expressed genes was represented in Venn diagrams. To determine gene expression changes that occur in PCa cells upon co-culture with CAFs, CAF-specific genes were subtracted. **c** Expression of HMGCS2 and AKR1C3 was validated by real-time RT-PCR in LNCaP and DuCaP monoculture spheroids and in LNCaP and DuCaP cells after spheroid co-culture with CAFs (LN/CAF, Du/CAF). Separation of cells was performed by flow cytometry-assisted cell sorting based on GFP expression in CAFs as described under methods. Values are expressed as relative expression of the gene of interest (GOI) normalized to hydroxymethylbilane synthase (HMBS). **d** Simplified overview of cholesterol and steroid biosynthesis metabolism. **e** Western blot analysis of HMGCS2 and AKR1C3 in mono vs co-culture spheroids. Glyceraldehyde 3-phosphate dehydrogenase (GAPDH) was used as loading control. Quantification of bands was performed with Image Studio (Li-Cor) and the ratio between the protein of interest and GAPDH was blotted in a graph. Data are represented as mean ± SEM from at least three independent experiments. (* *P* < 0.05, ** *P* < 0.01)

We first identified the gene expression profiles of LNCaP and DuCaP cells when grown as 3D spheroids and compared them to conventional 2D culture over 8 days. As shown in Fig. 1, 3D spheroid culture resulted in significant alteration of several hundreds of genes in both tumor cell lines (LNCaP 3Dvs2D: *n* = 347, DuCaP 3Dvs2D: *n* = 845) (Fig. 1a). A panel of 39 genes was similarly regulated in the two cancer cell lines (Table 2). Among these, 18 genes were significantly upregulated. These were annotated to cell adhesion and ECM-receptor interaction like fermitin family member 2 (FERMT2, also known as kindlin-2), plasminogen activator urokinase receptor (PLAUR), peroxisome proliferator-activated receptor gamma (PPARG) that plays a role in adipocyte differentiation, zyxin (ZYX), which is densely found at focal adhesions, and the anti-apoptotic gene ankyrin repeat and KH domain containing 1 (ANKHD1). In addition, genes related to oxidative stress and NFkB signaling (growth/differentiation factor 15, GDF-15, spermidine/spermine N1-acetyltransferase 1, SAT1, sequestome 1, SQSTM1, thyroid hormone receptor interacting protein 3, TRIP3) were upregulated in both cell lines upon 3D culture. By contrast, genes related to cell cycle and DNA replication such as cell division cycle 45-like (CDC45L), checkpoint kinase 1 (CHEK1), and thymidine kinase 1, soluble (TK1) were among the most significantly downregulated genes in 3D spheroids, suggesting that PCa cells exhibit strong cell-to-cell and cell-to-ECM interactions but a low proliferative activity in 3D compared to 2D culture.

**Table 2:** Genes similarly regulated in LNCaP and DuCaP 3D spheroids compared to 2D culture

	fold change 3D vs 2D		
Symbol	LNCaP	DuCaP	Name
ANKHD1	2.00	2.38	ankyrin repeat and KH domain containing 1
CCPG1	2.47	2.78	cell cycle progression 1
FERMT2	2.43	5.05	fermitin family member 2
GDF15	2.40	11.10	growth differentiation factor 15
HSPH1	2.13	2.31	heat shock protein family H, member 1
LARP6	3.82	2.51	La ribonucleoprotein domain family member 6
MYLIP	2.32	2.05	myosin regulatory light chain interacting protein
PLAUR	2.03	2.93	plasminogen activator, urokinase receptor
PPARG	2.15	11.33	peroxisome proliferator-activated receptor gamma
SAT1	2.41	3.07	spermidine/spermine N1-acetyltransferase 1
SH3GLB1	2.11	3.50	SH3-domain GRB2-like endophilin B1
SLC3A2	2.23	3.51	solute carrier family 3 (amino acid transporter heavy chain), member 2
SPIRE1	2.86	2,44	spire-type actin nucleation factor 1
SQSTM1	2.57	3.00	sequestosome 1
TRIB3	2.34	2.08	tribbles pseudokinase 3
UPP1	2.39	2.62	uridine phosphorylase 1
ZFAND2A	2.04	2.11	zinc finger, AN1-type domain 2A
ZYX	3.61	4.09	zyxin
CDC45L	0.17	0.45	cell division cycle 45
CHEK1	0.41	0.48	checkpoint kinase 1
E2F2	0.36	0.43	E2F transcription factor 2
EGR1	0.15	0.10	early growth response 1
FOS	0.10	0.04	FBJ murine osteosarcoma viral oncogene homolog
FOXM1	0.47	0.31	forkhead box M1
GINS2	0.25	0.44	GINS complex subunit 2 (Psf2 homolog)
H2AFX	0.46	0.38	H2A histone family member X
HMMR	0.18	0.49	hyaluronan mediated motility receptor
ID3	0.45	0.25	inhibitor of DNA binding 3, dominant negative helix-loop-helix protein
KIAA0101	0.29	0.48	KIAA0101
KIF11	0.44	0.49	kinesin family member 11
LOC731314	0.27	0.37	similar to H2A histone family, member X
NCAPD2	0.46	0.45	Condensin complex subunit 1
PFKFB4	0.43	0.45	6-phosphofructo-2-kinase/fructose-2,6-biphosphatase 4
SLC45A3	0.41	0.42	solute carrier family 45 member 3
TK1	0.30	0.44	thymidine kinase 1, soluble
TYMS	0.26	0.49	thymidylate synthetase
UHRF1	0.31	0.43	ubiquitin-like with PHD and ring finger domains 1
TPX2	0.44	0.40	TPX2, microtubule-associated
TUBA1A	0.37	0.46	tubulin alpha 1a

### Co-culture with CAFs induces an upregulation of cholesterol and steroid biosynthesis pathways in prostate cancer cells

We next looked at changes in gene expression, which occur in PCa epithelial cells upon co-culture with CAFs. In particular, we compared the gene expression profiles of co-culture spheroids (LN/CAF, Du/CAF) with those of monoculture spheroids (LNCaP, DuCaP) and excluded all “stromal” genes that were regulated in CAF 3D versus 2D culture (Fig. 1b). This analysis recovered 57 genes that were similarly regulated in the two cancer cell lines upon co-culture with CAFs (Table 3). Notably, these 57 genes were all significantly up-regulated and included genes like 3-hydroxy-3-methylglutaryl-CoA synthase 2 (HMGCS2), a central enzyme of the cholesterol/ketogenesis pathway, which was among the 5 most up-regulated genes in both cell lines (LN/CAF vs LNCaP: 5.4-fold, Du/CAF vs DuCaP: 19.8-fold), 24-dehydrocholesterol reductase (DHCR24), collagen, type VI, alpha 3 chain (COL6A3), isocitrate dehydrogenase 2 (NADP+) (IDH2), glutamate receptor, ionotropic, N-methyl D-aspartate-associated protein 1 (GRINA), signal transducer and activator of transcription 3 (STAT3), and matrix metallopeptidase 7 (MMP7) (Table 3). Noteworthy, nuclear factor of kappa light polypeptide gene enhancer in B-cells 1 (NFKB1) was increased in co-culture spheroids, presuming an inflammatory phenotype.

**Table 3:** Genes differentially expressed in Du/CAF (DuCaP co-cultured with CAFs) and LN/CAF (LNCaP co-cultured with CAFs) compared to DuCaP and LNCaP monoculture spheroids

Symbol	Du/CAF vs DuCaP	LN/CAF vs LNCaP	Name
	Fold change		
SERPINA3	87.87	9.26	Serpin peptidase inhibitor, clade A (alpha-1 antiproteinase, antitrypsin)
CFB	87.73	7.24	Complement factor B
FGL1	21.10	6.70	Fibrinogen-like 1 (FGL1)
PLA2G2A	20.41	2.83	Phospholipase A2, group IIA (platelets, synovial fluid)
HMGCS2	19.79	5.43	3-hydroxy-3-methylglutaryl-Coenzyme A synthase 2 (mitochondrial)
HLA-DRA	14.19	2.50	Major histocompatibility complex, class II, DR alpha
COL6A3	11.12	2.81	Collagen, type VI, alpha 3
IL32	11.07	3.80	interleukin 32 (IL32), transcript variant 4, mRNA.
TGFBI	10.72	3.04	Transforming growth factor, beta-induced, 68 kDa
IRF1	9.80	2.73	Interferon regulatory factor 1
TAPBP	9.45	2.88	TAP binding protein (tapasin)
SCNN1A	9.06	2.14	Sodium channel, nonvoltage-gated 1 alpha
MMP7	8.97	2.57	Matrix metallopeptidase 7 (matrilysin, uterine)
STAT3	8.90	3.05	Signal transducer and activator of transcription 3 (acute-phase response factor)
TAP1	5.08	2.41	Transporter 1, ATP-binding cassette, sub-family B (MDR/TAP)
JUNB	4.81	2.19	Jun B proto-oncogene
COL3A1	4.62	2.09	Collagen, type III, alpha 1
MALL	4.43	2.81	Mal, T-cell differentiation protein-like
DDR1	4.15	2.05	Discoidin domain receptor tyrosine kinase 1
CPB1	3.78	2.88	Carboxypeptidase B1 (tissue)
CGN	3.60	2.21	Cingulin
GRHL2	3.46	2.46	Grainyhead-like 2 (Drosophila)
CD276	3.34	2.22	CD276 molecule
CYP2J2	3.31	2.59	Cytochrome P450, family 2, subfamily J, polypeptide 2
GPER	3.24	2.60	G protein-coupled estrogen receptor 1
GRINA	3.23	2.28	Glutamate receptor, ionotropic, N-methyl D-aspartate-associated protein 1 (glutamate binding)
UBE1	3.09	2.26	Ubiquitin-activating enzyme E1
VWA1	3.06	2.01	von Willebrand factor A domain containing 1
NFKB1	3.04	2.30	Nuclear factor of kappa light polypeptide gene enhancer in B-cells 1
HIPK2	2.92	2.09	Homeodomain interacting protein kinase 2
ATP1A1	2.85	2.60	ATPase, Na+/K+ transporting, alpha 1 polypeptide
ABCC5	2.80	2.09	ATP-binding cassette, sub-family C (CFTR/MRP), member 5
EIF4G1	2.78	2.74	Eukaryotic translation initiation factor 4 gamma, 1
TMEM79	2.74	2.32	Transmembrane protein 79
FRMD8	2.74	2.08	FERM domain containing 8
ALDH3B2	2.73	2.29	Aldehyde dehydrogenase 3 family, member B2
ANKFY1	2.71	2.08	Ankyrin repeat and FYVE domain containing 1
MAP 1B	2.62	2.51	Microtubule-associated protein 1B
LRG1	2.60	2.07	Leucine-rich alpha-2-glycoprotein 1
MYH9	2.54	2.02	Myosin, heavy chain 9, non-muscle
DHCR24	2.54	2.35	24-dehydrocholesterol reductase
CANT1	2.49	2.06	Calcium activated nucleotidase 1
UBA1	2.43	2.09	Tubulin, alpha 1a
IDH2	2.42	2.40	Isocitrate dehydrogenase 2 (NADP+) mitochondrial, nuclear gene encoding mitochondrial protein
RTN1	2.38	2.03	Reticulon 1
DHCR7	2.31	2.23	7-dehydrocholesterol reductase
VCP	2.31	2.52	Valosin-containing protein
MVP	2.23	2.05	Major vault protein
PROM2	2.19	2.21	Prominin 2
DTX2	2.16	2.11	Deltex homolog 2 (Drosophila)
UBE2G1	2.11	2.01	Ubiquitin-conjugating enzyme E2G 1 (UBC7 homolog, yeast)
DYNC1H1	2.11	2.30	Dynein, cytoplasmic 1, heavy chain 1
TPR	2.08	2.12	Translocated promoter region (to activated MET oncogene)
TGFBR3	2.08	3.76	Transforming growth factor, beta receptor III
EIF2C2	2.08	2.02	Eukaryotic translation initiation factor 2C, 2
EPHX1	2.07	2.60	Epoxide hydrolase 1, microsomal (xenobiotic)
PLEKHF1	2.06	2.48	Pleckstrin homology domain containing, family F (with FYVE domain) member 1

Based on our previous study where enzalutamide resistance was more prominent in Du/CAF than in LN/CAF co-culture spheroids [11], we further looked at genes, which were particularly altered in DuCaP cells upon co-culture with CAFs. We found 1295 differentially regulated genes with “steroid hormone biosynthesis” among the top ranked differentially expressed pathways based on KEGG pathway analysis (Table 4). A more detailed interrogation of the data revealed a significant up-regulation of genes that are involved in cholesterol synthesis (DHCR7, DHCR24, SC4MOL, SC5DL), aldo-keto reductase family genes (AKR1C3, AKR1C4), which mediate the conversion of adrenal androgens into the more active androgens testosterone and dihydrotestosterone, and UDP glucuronosyltransferase family genes (UGT1A1, UGT2B7, UGT2B10, UGT2B17), which play a role in the conjugation and subsequent elimination of endogenous compounds like estrogens (Table 5). Overall, these data suggest that CAFs induce an upregulation of cholesterol and steroid biosynthesis pathways in PCa cells.

**Table 4:** KEGG pathways with XD scores > 0.96 in Du/CAF vs DuCaP spheroids

Annotation (pathway/process)	XD score
hsa00053: Ascorbate and aldarate metabolism	1.891
hsa00040: Pentose and glucuronate interconversions	1.705
hsa00360: Phenylalanine metabolism	1.621
hsa00100: Steroid biosynthesis	1.489
hsa00140: Steroid hormone biosynthesis	1.371
hsa00980: Metabolism of xenobiotics by cytochrome p450	1.252
hsa04966: Collecting duct acid secretion	1.171
hsa00982: Drug metabolism - cytochrome P450	1.092
hsa00860: Porphyrin and chlorophyll metabolism	1.085
hsa00511: Other glycan degradation	1.059
hsa00120: Primary bile acid biosynthesis	1.059
hsa00500: Starch and sucrose metabolism	1.024
hsa00983: Drug metabolism - other enzymes	0.991

**Table 5:** Differentially regulated genes annotated to steroid biosynthesis in Du/CAF co-culture spheroids

Symbol	Fold change	Name
AKR1C4	6.77	Aldo-keto reductase family 1, member C4
HSD11B2	5.37	Hydroxysteroid (11-beta) dehydrogenase 2
UGT1A1	5.22	UDP glucuronosyltransferase 1 family, polypeptide A1
AKR1C3	4.06	Aldo-keto reductase family 1, member C3
UGT1A3	3.04	UDP glucuronosyltransferase 1 family, polypeptide A3
DHCR7	2.31	7-dehydrocholesterol reductase
DHCR24	2.54	24-dehydrocholesterol reductase
LSS	2.34	Lanosterol synthase (2,3-oxidosqualene-lanosterol cyclase)
HSD17B7	2.11	Hydroxysteroid (17-beta) dehydrogenase 7
SC4MOL	2.41	Sterol-C4-methyl oxidase-like
SC5DL	2.60	Sterol-C5-desaturase (ERG3 delta-5-desaturase homolog, S. cerevisiae)-like
UGT2B11	2.31	UDP glucuronosyltransferase 2 family, polypeptide B11
CYP3A5	2.61	Cytochrome P450, family 3, subfamily A, polypeptide 5
HSD17B7	2.11	Hydroxysteroid (17-beta) dehydrogenase 7
UGT2B7	2.74	UDP glucuronosyltransferase 2 family, polypeptide B7
UGT2B10	2.57	UDP glucuronosyltransferase 2 family, polypeptide B10
UGT2B17	2.53	UDP glucuronosyltransferase 2 family, polypeptide B17
UGT1A6	0.42	UDP glucuronosyltransferase 1 family, polypeptide A6

### HMGCS2 and AKR1C3 are significantly upregulated in PCa cells upon co-culture with CAFs

We next validated the expression of CAF-induced differentially regulated genes in LNCaP and DuCaP cells through real-time RT-PCR. To this end, we separated the tumor cells from the CAFs after spheroid co-culture using flow-cytometry assisted cell sorting based on GFP expression in CAFs. Two genes, HMGCS2 and AKR1C3, were confirmed to be significantly up-regulated in co-culture versus monoculture spheroids with a more pronounced upregulation in Du/CAF compared to LN/CAF. Their expression in CAFs on the other hand was absent or only very weak (Fig. 1c). HMGCS2 and AKR1C3 are part of the cholesterol and steroid biosynthesis pathways, respectively (Fig. 1d). HMGCS2 is a mitochondrial enzyme that condenses acetyl CoA and acetoacetyl CoA to hydroxyl-methylglutaryl CoA (HMG-CoA), which is further transformed into the ketone bodies acetoacetate, hydroxybutyrate and acetone [19]. AKR1C3 (also named type 5 17ß-hydroxysteroid dehydrogenase, 17ßHSD5) plays an important role in steroid biosynthesis by mediating the conversion of adrenal androgens into the active androgen testosterone [20]. CAF-induced upregulation of HMGCS2 and AKR1C3 was further confirmed on protein level through Western blotting. As shown in Fig. 1e, HMGCS2 and AKR1C3 were elevated in co-culture spheroids compared to monoculture spheroids and their expression was weak to absent in CAFs.

### Upregulation of HMGCS2 and AKR1C3 in tumor cells through CAF-conditioned medium

**Fig. 2:**
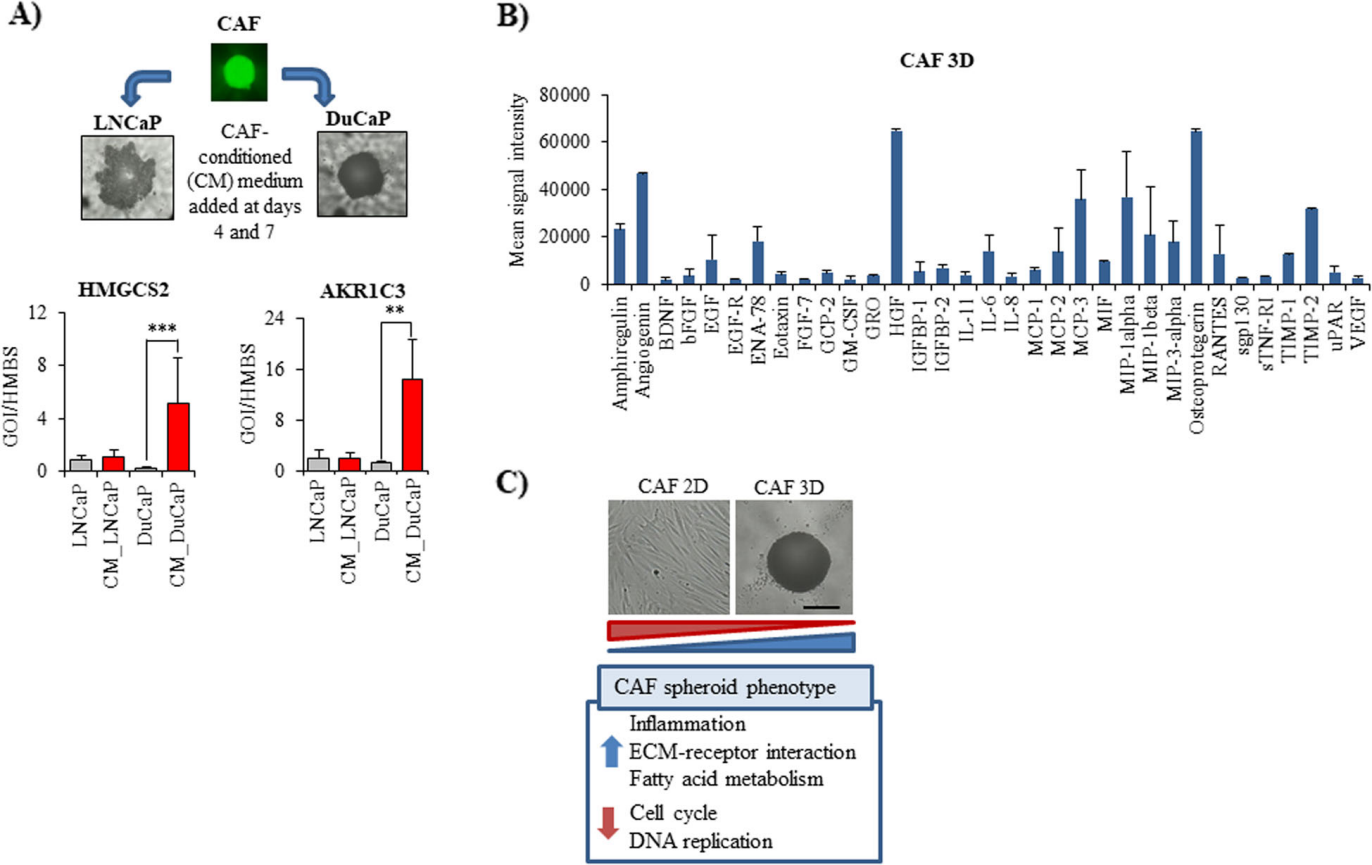
HMGCS2 and AKR1C3 expression is increased in PCa cells by incubation with CAF-conditioned medium. **a** mRNA expression of HMGCS2 and AKR1C3 was analyzed in LNCaP and DuCaP cells after 8 days of 3D spheroid culture in CAF-conditioned (CM) medium and compared to cells, which were cultured in standard medium. HMBS was used as internal control. Data represent the mean plus SEM from at least three independent experiments. (* *P* < 0.05, ** *P* < 0.01,*** *P* < 0.001) (**b**) Conditioned medium of CAF 3D spheroids was loaded onto a semi-quantitative RayBio® Human Cytokine Antibody Array (G-Series 1000, RayBiotech, Norcross, GA). Values were normalized to culture medium and expressed as mean signal intensity with SEM from three independent experiments. **c** CAFs were cultured either in T75 flasks (2D) or in 96 well hanging drop plates at 8000 cells per well. Alterations in mRNA gene expression were determined via Illumina microarray analysis. Significantly altered pathways between 2D and 3D cultured CAFs were identified via KEGG analysis. Representative phase contrast images are shown for CAFs grown either in 75cm^2^ culture flasks (2D) or as 3D spheroids in 96 well hanging drop plates (magnification 10x, scale bar: 500 μm)

We then analyzed whether CAF-induced upregulation of HMGCS2 and AKR1C3 was due to paracrine signaling between tumor cells and CAFs. Hence, we cultured LNCaP and DuCaP cells in 96 well hanging drop plates to form spheroids and then treated them with CAF spheroid-conditioned medium over 8 days. As shown in Fig. 2a, HMGCS2 and AKR1C3 were significantly increased in DuCaP spheroids after culture in CAF-conditioned medium, suggesting that a paracrine interaction of the two cell types is sufficient for the upregulation of HMGCS2 and AKR1C3. In LNCaP cells, expression of HMGCS2 and AKR1C3 did not significantly change upon treatment with CAF-conditioned medium.

To better understand the paracrine interaction between tumor epithelial cells and CAFs, we next performed a cytokine profiling in the supernatant of CAF 3D spheroids. As shown in Fig. 2b, CAFs secreted high amounts of inflammatory cytokines and chemokines like amphiregulin, angiogenin, ENA-78 (also known as CXC motif chemokine ligand 5), HGF (hepatocyte growth factor), IL-6 (interleukin-6), MCP-3 (monocyte-chemotactic protein 3, also known as chemokine ligand 7, CCL7), MIP-1 alpha (macrophage inflammatory protein-1, CCL3), osteoprotegerin, RANTES (regulated on activation, normal T cell expressed and secreted, CCL5), and TIMP (tissue inhibitor of metalloproteinases)-1, − 2. Correspondingly, microarray mRNA analysis revealed a panel of inflammatory genes such as interleukin-1ß, interleukin 11, interleukin 24, interleukin-6, interleukin-8, and chemokine (C-X-C motif) ligand 5, which were significantly up-regulated in CAFs upon 3D culture (Table 6). Besides, microarray profiling revealed that several cell cycle and DNA replication genes were significantly downregulated in CAF 3D spheroids, suggesting a lower proliferative activity in 3D compared to 2D culture (Fig. 2c). These data confirm the findings of our previous study where CAFs were shown to exhibit a lower proliferative activity in 3D culture [11]. Among the significantly upregulated pathways, we identified “ECM-receptor interaction”, including genes encoding for tenascin C, laminin beta 3, collagen type IV (alpha 1, 2, and 6), integrin alpha 2 (CD49B) and versican (VCAN), a large ECM proteoglycan, that was among the 10 most up-regulated genes in CAFs upon spheroid culture (Table 6). In addition, we found major changes in the fatty acid metabolism pathway of CAF spheroids. In particular, three genes encoding enzymes that are required for the synthesis and oxidation of long-chain fatty acids (ACSL 1 and 4, acyl-CoA synthetase long-chain family member 1 and 4, ACADVL, acyl-CoA dehydrogenase, very long chain) were significantly up-regulated in CAF 3D spheroids compared to 2D culture. Overall, these data suggest that CAFs grown under 3D culture conditions acquire a low proliferative but strong inflammatory phenotype.

**Table 6:** Genes differentially regulated in 3D CAF spheroids compared to 2D culture

Inflammation	
IL1B	Interleukin 1, beta
PTGS2	Prostaglandin-endoperoxide synthase 2
CXCL5	Chemokine (C-X-C motif) ligand 5
IL11	Interleukin 11
IL24	Interleukin 24
CXCL2	Chemokine (C-X-C motif) ligand 2
IL13RA2	Interleukin 13 receptor, alpha 2
IL6	Interleukin 6
CCL20	Chemokine (C-C motif) ligand 20
IL24	Interleukin 24
IL8	Interleukin 8
IL1A	Interleukin 1, alpha
IL33	Interleukin 33
CCL7	Chemokine (C-C motif) ligand 7
IRAK2	Interleukin-1 receptor-associated kinase 2
IL18R1	Interleukin 18 receptor 1
IL1R1	Interleukin 1 receptor, type I
TNFAIP3	Tumor necrosis factor, alpha-induced protein 3
CCRL1	Chemokine (C-C motif) receptor-like 1
LOC651872	C-C chemokine receptor type 11 (C-C CKR-11)
ISG20	Interferon stimulated exonuclease gene 20 kDa
CXCL1	Chemokine (C-X-C motif) ligand 1
IL4R	Interleukin 4 receptor
TNFAIP6	Tumor necrosis factor, alpha-induced protein 6
IL1RN	Interleukin 1 receptor antagonist
INSIG1	Insulin induced gene 1
TNFSF10	Tumor necrosis factor (ligand) superfamily, member 10
CCL3L1	Chemokine (C-C motif) ligand 3-like 1
PTGER4	Prostaglandin E receptor 4 (subtype EP4)
IL1F9	Interleukin 1 family, member 9
CCL3L1	Chemokine (C-C motif) ligand 3-like 1
IL7R	Interleukin 7 receptor
IL23A	Interleukin 23, alpha subunit p19
IFRD1	Interferon-related developmental regulator 1
INSIG2	Insulin induced gene 2
ECM-receptor interaction	
TNC	Tenascin C (hexabrachion)
LAMB3	Laminin, beta 3
THBS2	Thrombospondin 2
COL4A6	Collagen, type IV, alpha 6
COL4A1	Collagen, type IV, alpha 1
ITGA2	Integrin, alpha 2 (CD49B)
COL4A2	Collagen, type IV, alpha 2
Fatty acid metabolism	
ACSL4	Acyl-CoA synthetase long-chain family member 4
ACADVL	Acyl-Coenzyme A dehydrogenase, very long chain
ACSL1	Acyl-CoA synthetase long-chain family member 1

### AKR1C3 and HMGCS2 expression is associated with diminished response of prostate cancer cells to AR targeted therapies

**Fig. 3:**
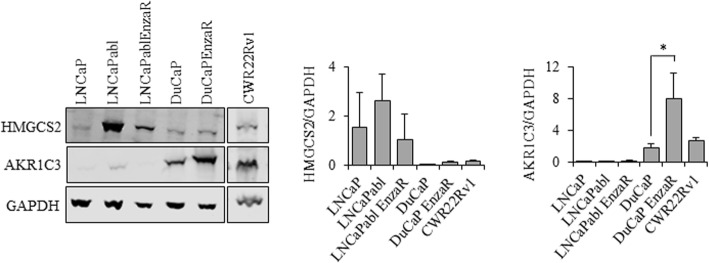
Representative Western blots of HMGCS2 and AKR1C3 in various PCa cell lines after conventional 2D culture. Enzalutamide resistant sublines (EnzaR) were established through long-term culture of parental cells in the presence of 8 μM enzalutamide. Quantification of bands was performed with Image Studio (Li-Cor) and the ratio between the protein of interest and GAPDH was blotted in a graph. Data represent the mean plus SEM from at least three independent experiments. (* *P* < 0.05, ** *P* < 0.01,*** *P* < 0.001)

To test whether the two selected genes play a role in AR targeted therapy resistance, we next investigated the expression of HMGCS2 and AKR1C3 in various PCa cell lines mimicking CRPC and/or enzalutamide resistance. As shown in Fig. 3, the overall expression of the two enzymes was heterogeneous among the cell lines. Remarkably, HMGCS2 expression was strongest in LNCaPabl cells, a subline mimicking CRPC, suggesting that HMGCS2 expression might play a role in progression to CRPC. However, HMGCS2 was not increased in the enzalutamide-resistant PCa cell lines, DuCaP EnzaR and LNCaPabl EnzaR, and also not in CWR22Rv1 (Fig. 3), which have been previously reported as enzalutamide resistant [21]. AKR1C3, on the other hand, was weakly expressed in LNCaPabl cells but substantially expressed in DuCaP, DuCaP EnzaR and was also weak in CWR22Rv1 cells. Strongest expression was observed in DuCaP EnzaR and CWR22Rv1, indicating that AKR1C3 might be associated with enzalutamide resistance.

### Increased expression of HMGCS2 and AKR1C3 clinical human prostate cancer specimens

**Fig. 4:**
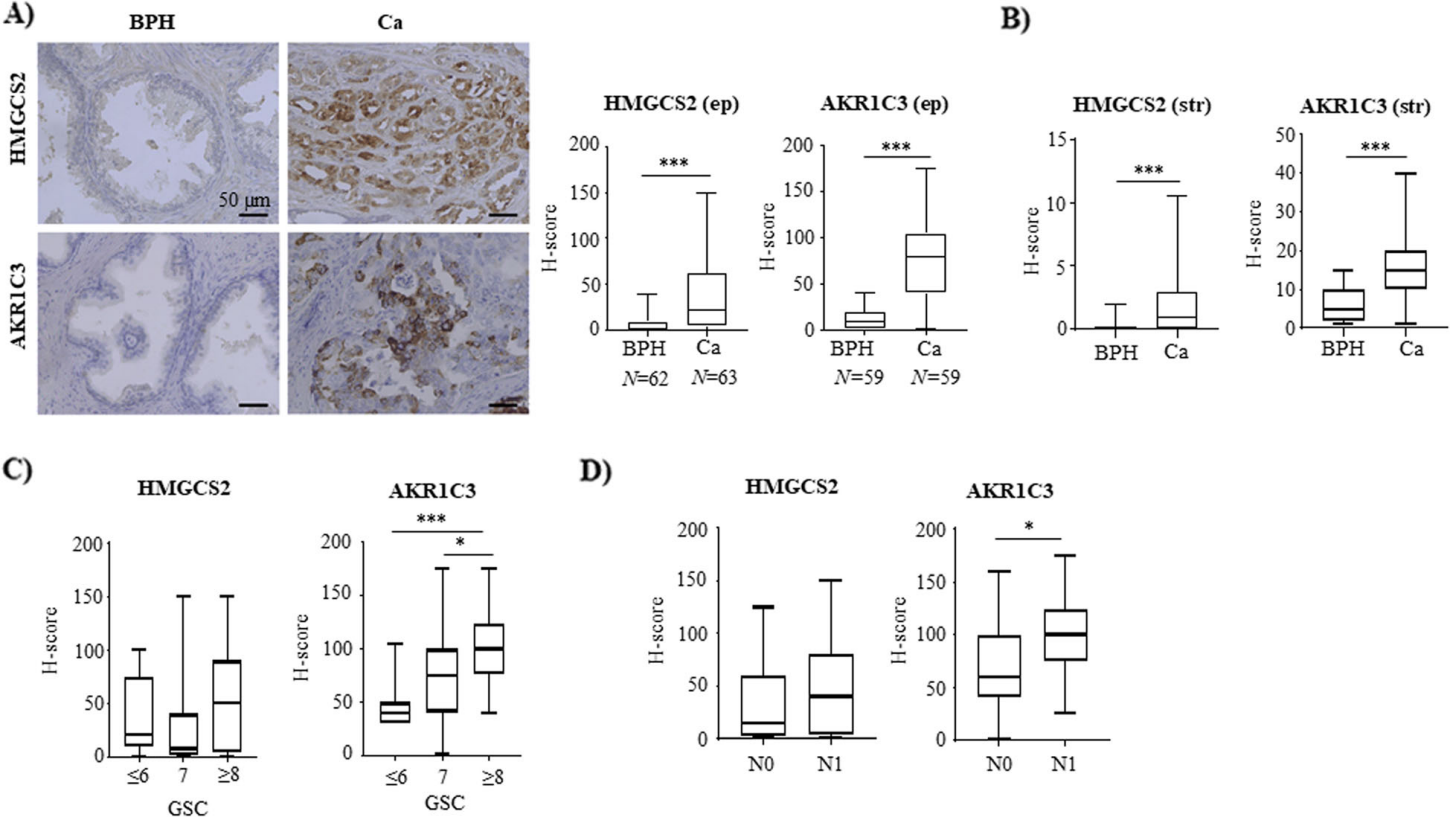
HMGCS2 and AKR1C3 expression is significantly increased in human PCa. **a, b** Representative staining for HMGCS2 and AKR1C3 in benign (BPH) prostate tissue and PCa. Staining intensity was quantified by determining H-score in the epithelium (ep) (**a**) and in the stroma (str) (**b**) as described under methods. **c** Expression of HMGCS2 and AKR1C3 was correlated with Gleason score (GSC) (GSC ≤ 6: *N* = 12, GSC = 7: *N* = 31, GSC ≥ 8: *N* = 19) and (**d**) lymph node metastases (N0: *N* = 41, N1: *N* = 20). Data are represented as mean + SEM. (* *P* < 0.05, *** *P* < 0.001)

To assess the impact of HMGCS2 and AKR1C3 in patient samples, we looked at their expression in human prostate tissue by immunohistochemistry. In a cohort of 69 patients, both, HMGCS2 and AKR1C3, were predominantly expressed in epithelial cells with significantly higher expression in PCa compared to benign tissue (Fig. 4a). Both enzymes were also expressed in the stroma although with much lesser extent (Fig. 4b). Despite this low expression, however, we observed that - also in the stromal compartment - HMGCS2 and AKR1C3 were significantly higher in cancer-associated compared to benign areas. AKR1C3 expression also correlated with Gleason grade (Fig. 4c) and the presence of metastatic lymph nodes (N-stage) (Fig. 4d). There was also a trend towards higher HMGCS2 staining intensity in PCa with a Gleason grade of ≥8 as well as in patients with positive lymph nodes, although the differences were not statistically significant (Fig. 4c, d).

### HMGCS2 expression significantly affects cell viability and spheroid growth of castration resistant LNCaPabl cells

**Fig. 5:**
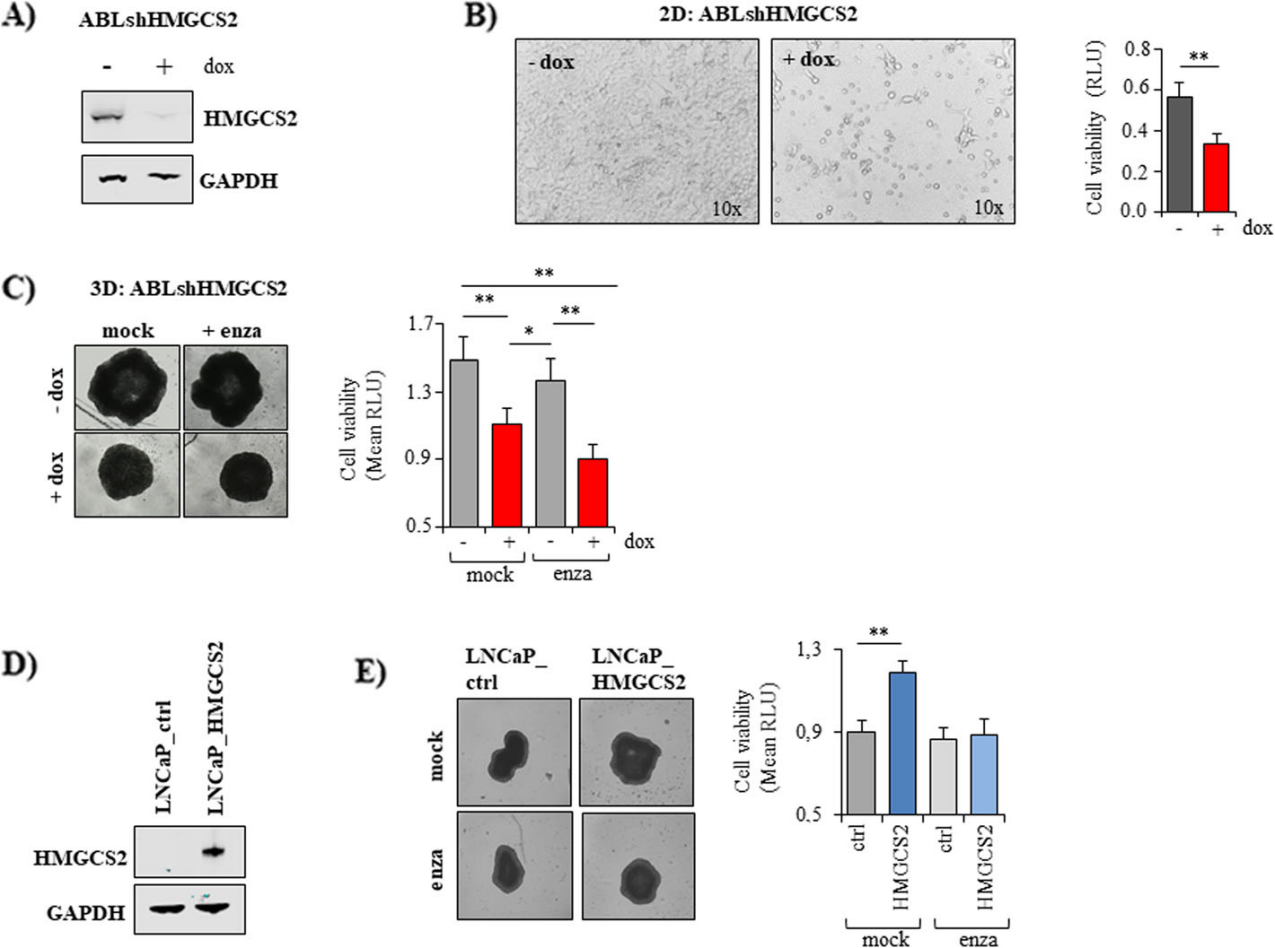
Effects of HMGCS2 knockdown and overexpression on PCa cell growth. **a** LNCaPabl cells were stably infected with a doxycycline-inducible shHMGCS2 vector (ABLshHMGCS2). Following treatment with 1 μM doxycycline, HMGCS2 was effectively downregulated on protein level compared to the mock control as shown by Western analysis. GAPDH was used as loading control. **b** ABLshHMGCS2 cells were seeded into 96 well plates and incubated in the absence or presence of doxycycline over 5 days. Cell viability was determined with CellTiterGlo viability assay (Promega). Representative images were taken at the end of treatment (magnification 10x). **c** ABLshHMGCS2 cells were seeded into ULC 96 well plates (Corning) and allowed to form spheroids over 4 days. Then, 1 μM doxycycline (dox) and 5 μM enzalutamide (enza) were added. Cell viability was determined through CellTiterGlo viability assay after 10 days of treatment. Medium was exchanged twice a week. Representative images were taken at day 10 with IncuCyte S3 software. **d** LNCaP cells were transiently transfected with a HMGCS2 plasmid (LNCaP_HMGCS2). HMGCS2 overexpression was confirmed 72 h afterwards by Western blotting. GAPDH was used as internal control. **e** LNCaP cells were transiently transfected with a HMGCS2 plasmid and seeded into a 96 well ULC plate (Corning) to allow 3D spheroid formation. After 4 days, 5 μM enzalutamide was added in RPMI with 10% CS_FCS. After 10 days, cell viability was measured via CellTiterGlo assay. Representative images were taken at the end of treatment with IncuCyte S3 software. Data represent the mean plus SEM from at least three independent experiments. (* *P* < 0.05, ** *P* < 0.01)

To further elucidate the role of HMGCS2 in PCa cells, we generated a doxycycline-inducible lentiviral specific shHMGCS2 vector that was expressed in LNCaPabl cells, which exhibit strong HMGCS2 expression. Efficient downregulation of HMGCS2 in the presence of doxycycline was confirmed by Western blotting (Fig. 5a). Knockdown of HMGCS2 was associated with significantly reduced cell growth in 2D culture (Fig. 5b) and significantly impaired 3D spheroid growth (Fig. 5c). However, HMGCS2 knockdown did not significantly increase the growth-inhibitory effects of enzalutamide.

We then transiently overexpressed HMGCS2 in androgen-responsive LNCaP cells, which hardly express HMGCS2 (Fig. 5d). As shown in Fig. 5e, this ectopic expression of HMGCS2 significantly increased LNCaP spheroid growth (Fig. 5e). However, HMGCS2 overexpression did not render the cells less responsive to the antiandrogen enzalutamide, suggesting that HMGCS2 alone does not play an essential role in enzalutamide resistance.

### Inhibition of cholesterol synthesis with simvastatin significantly inhibits castration and enzalutamide-resistant cells

**Fig. 6:**
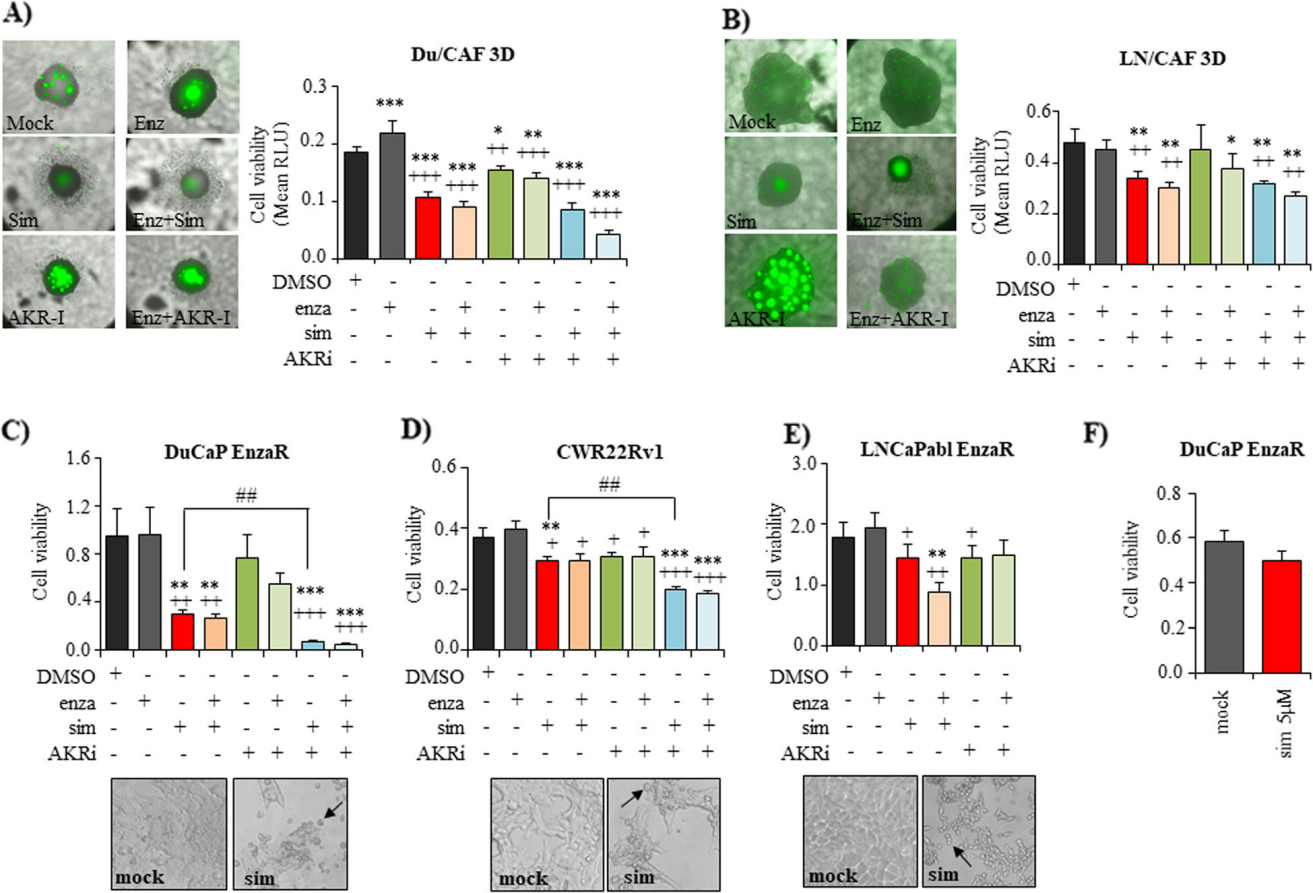
Combined blockade of cholesterol and steroid biosynthesis with simvastatin and AKRi to inhibit PCa cells. Du/CAF (**a**) and LN/CAF (**b**) co-culture spheroids were established as described under methods. CAFs stably express GFP to distinguish them from tumor cells. Four days after seeding, spheroids were treated with 5 μM enzalutamide (enza), 5 μM simvastatin (sim), and 50 μM of an inhibitor against AKR1C3 (AKRi) in medium supplemented with 10% CS_FCS. Cell viability was assessed after 10 days using CellTiterGlo assay. (**c, d, e**) DuCaP EnzaR, CWR22Rv1, and LNCaPabl EnzaR were seeded into 96 well plates. After overnight incubation, drugs were added in medium with 10% CS_FCS as indicated. Representative images were taken after 6 days of treatment with 5 μM simvastatin and cropped to show typical rounded cells that were depicted with a black arrow. Cell viability was determined after 6 days via CellTiterGlo cell viability assay and expressed as mean relative luminescence units (RLU) from at least 3 independent experiments with SEM. **f** DuCaP EnzaR were seeded into 96 well plates. Treatment with 5 μM simvastatin was performed in RPMI + 10% FCS. Cell viability was determined after 6 days of treatment via CellTiterGlo cell viability assay and expressed as mean relative luminescence units (RLU) from at least three independent experiments with SEM. (* and ^+^ *P* < 0.05, ** and ^++^ *P* < 0.01, *** and ^+++^ *P* < 0.001, * related to mock control, ^+^ related to enzalutamide-treated cells)

Since KEGG pathway analysis of microarray data identified cholesterol metabolism (including HMGCS2) as one of the most significantly up-regulated pathways, we further investigated the effects of inhibiting cholesterol synthesis by simvastatin. Simvastatin is a frequently prescribed cholesterol-lowering drug that intervenes with HMG CoA reductase (HMGCR) within the mevalonate pathway. As shown in Fig. 6, 5 μM simvastatin significantly inhibited spheroid growth of Du/CAF (Fig. 6a) and LN/CAF (Fig. 6b) co-cultures under androgen-deprived conditions (10% CS-FCS). In addition, enzalutamide resistant DuCaP EnzaR (Fig. 6c), castration and enzalutamide-resistant CWR22Rv1 (Fig. 6d) and LNCaPabl EnzaR (Fig. 6e) cells were significantly inhibited in growth after treatment with simvastatin in 2D culture, suggesting that blocking cholesterol synthesis could overcome AR targeted therapy resistance. Notably, treatment with simvastatin induced a typical rounding-up of the cells. Moreover, we noticed that the growth-inhibitory effect of simvastatin was considerably impaired in the presence of 10% FCS as demonstrated in DuCaP EnzaR cells (Fig. 6).

### Dual targeting of cholesterol and steroid biosynthesis significantly inhibits therapy-resistant prostate cancer cells

Since microarray analysis revealed both, cholesterol metabolism and steroid biosynthesis, as significantly upregulated in co-culture spheroids, we hypothesized that simultaneous targeting of both pathways might have the strongest anti-proliferative effect. In previous studies, increased AKR1C3 expression has been associated with CRPC [22] and enzalutamide-resistance [21]. Moreover, inhibiting the enzymatic activity of AKR1C3 with indomethacin was shown to reduce tumor growth in vitro and in vivo and re-sensitized cells to enzalutamide [21]. In this study, we used a specific AKR1C3 inhibitor (AKRi), which significantly inhibited growth of Du/CAF spheroid co-cultures but not that of LN/CAF. However, when we combined AKRi with the antiandrogen enzalutamide, both Du/CAF and LN/CAF spheroids were significantly inhibited in growth (Fig. 6a, b). AKRi also inhibited growth of the therapy-resistant cell lines DuCaP EnzaR, LNCaPabl EnzaR and CWR22Rv1 (Fig. 6c, d, e). Importantly, AKRi was able to significantly enhance the effect of simvastatin in enzalutamide resistant DuCaP EnzaR and CWR22Rv1 cells (Fig. 6c, d), indicating that combined targeting of cholesterol and steroid biosynthesis represents a promising way to inhibit AR targeted therapy resistant PCa cells.

## Discussion

The development of novel AR targeted drugs including enzalutamide has significantly improved therapy of CRPC, however, still the development of resistances is one of the major problems in the clinical management of PCa patients [23, 24]. Although several possible escape mechanisms have been identified in the past, including AR variant expression, elevated expression of glucocorticoid receptor, and overexpression of AKR1C3 [25], the reasons for the development of antiandrogen resistances are not yet fully understood. For that reason, this study aimed at looking for novel targets that render PCa cells therapy resistant. To this end, we used a previously established 3D co-culture model consisting of PCa cells and CAFs [11]. In this model, we could demonstrate that PCa cells become resistant to the antiandrogen enzalutamide when co-cultured with CAFs. Here, we identified gene expression changes, which occur in LNCaP and DuCaP cells upon 3D spheroid culture in the absence or presence of CAFs. Microarray analysis revealed that PCa cells acquire a typical gene expression profile in 3D culture with high expression of cell adhesion and ECM-receptor interaction genes and a low expression of genes annotated to cell cycle and DNA replication. Even more important, we found that PCa cells significantly upregulate cholesterol metabolism and steroid biosynthesis when grown as 3D co-culture spheroids in the presence of CAFs. In particular, we identified two genes, HMGCS2 and AKR1C3, which were significantly upregulated in PCa cells upon co-culture with CAFs on mRNA and protein level. Notably, incubation of tumor cells with CAF-conditioned medium alone also resulted in an upregulation of HMGCS2 and AKR1C3 suggesting a paracrine communication between tumor epithelial cells and CAFs. Notably, CAF-conditioned medium contained high amounts of inflammatory cytokines including IL-6. Concomitantly, we found a significant upregulation of various pro-inflammatory genes in CAF 3D spheroids. A recent study published by Patel and colleagues showed that IL-6 can increase cellular cholesterol uptake thereby mediating steroid synthesis under androgen deprived conditions. In addition, these authors suggested that pro-inflammatory cytokines may stimulate lipolysis in the tumor microenvironment that drives the formation of cholesterol [26]. Furthermore, CAF spheroids secreted substantial levels of angiogenin, HGF and osteoprotegerin, which have been previously associated with angiogenesis, migration/invasion and bone metastasis of PCa cells [27–30]. In summary, we suggest that CAFs mediate a dysregulation of cholesterol and steroid metabolism in PCa cells through a panel of pro-inflammatory, pro-migratory and pro-angiogenic cytokines and chemokines. Further studies are warranted to delineate the key players of this paracrine interaction which might also drive the cells into therapy resistance. With regard to this, however, it should be considered that the tumor-associated stroma comprises a highly heterogeneous mixture of various CAF subtypes, which may exhibit different effects on tumor cells from growth-promoting to growth-inhibiting ones [31, 32]. In addition, the amount of stroma within and around the tumor area is strongly varying among patients [33]. Hence further investigations using primary CAFs with clearly defined subtypes and varying ratios of CAFs to tumor cells are warranted.

Notably, this study showed that HMGCS2 and AKR1C3 were also elevated in human PCa specimens compared to benign tissue. While there are numerous previous studies, which have demonstrated elevated expression of AKR1C3 in late stage PCa correlating with Gleason score, CRPC and enzalutamide resistance [21, 34, 35], there are only few studies, which have linked HMGCS2 with PCa. This enzyme regulates the production of ketone bodies in the mitochondria [19]. Ketone bodies can be used by cells as compensatory energy sources during fast tumor growth [36] and converted to acetyl CoA and transduced to Krebs cycle (reviewed by [37]) or further used for cholesterol synthesis [38, 39]. Saraon and colleagues showed that HMGCS2 was about 9-fold higher in LNCaPabl compared to parental LNCaP cells, suggesting a link between elevated expression of HMGCS2 and CRPC [40]. These findings correspond with our data, which revealed highest expression of HMGCS2 in castration-resistant LNCaPabl cells. Knocking down HMGCS2 in LNCaPabl resulted in significantly reduced cell viability and diminished spheroid growth. Moreover, ectopic overexpression of HMGCS2 in LNCaP cells, on the other hand, significantly increased spheroid growth, suggesting a critical role of this enzyme in PCa. Though our data suggest that HMGCS2 plays a role in CRPC, we did not detect an upregulation of HMGCS2 expression in the enzalutamide-resistant PCa cell lines. Moreover, stable knockdown or ectopic overexpression of HMGCS2 did not change the cells` response to the antiandrogen enzalutamide.

Noteworthy, the impact of cholesterol metabolism in PCa has already been investigated in numerous studies. Recently, Yue et al. showed that PCa cells exhibit higher cholesterol levels through accumulating cholesteryl ester within lipid droplets [41]. This accumulation of cholesteryl ester was associated with impaired cholesterol efflux due to hyper-methylation of the cholesterol efflux transporter ABCA1 (ATP-binding cassette, sub-family A, member 1) [42] and enhanced AKT signaling [43]. In line with this, we have previously observed increased AKT signaling in LN/CAF co-culture spheroids [11]. Differences in AKT signaling due to a loss of the tumor suppressor PTEN (phosphatase and tensin homolog) in LNCaP cells may also be a possible reason that CAF-induced effects in this study were more pronounced in DuCaP cells, which express a functional PTEN. Though further studies are needed to clarify if and how AKT signaling influences CAF-induced cholesterol metabolism.

Epidemiologic studies have revealed that high serum cholesterol is associated with a higher risk of high-grade PCa [44]. Moreover, two recent clinical trials demonstrated that statins prolong the time to disease progression in patients with advanced PCa treated with ADT [45, 46]. Statins inhibit the rate-limiting step of endogenous cholesterol synthesis within the mevalonate pathway by targeting HMGCR and are widely used drugs to treat hypercholesteremia. In vitro, simvastatin was shown to enhance the effect of enzalutamide in LNCaP and VCaP cells [47]. Our group has previously reported on downregulation of the AR and its activity through statins [48]. Another study has demonstrated that statins cause a significant reduction in PSA levels [49]. In fact, our study showed that targeting the mevalonate pathway with simvastatin strongly and significantly inhibited cell growth of castration and enzalutamide resistant cells as well as 3D co-culture spheroid growth. Following simvastatin treatment, cells exhibited a typical rounded-up cell shape, which is most probably due to reduced cell membrane cholesterol levels.

Overall, the growth-inhibitory effect of simvastatin was much more potent than that of a specific inhibitor against AKR1C3, although AKR1C3 was significantly increased in enzalutamide resistant cells compared to their enzalutamide-responsive counterparts. These data confirm previous studies where AKR1C3 expression was correlated with enzalutamide resistance [21]. Notably, we used a specific inhibitor against AKR1C3 in our study which has only weak inhibitory side effects on cyclooxygenase I and II according to the manufacturer’s instructions. This may also explain the relatively weak inhibitory effect compared to indomethacin, a drug that has previously been shown to significantly inhibit PCa growth in vitro and in vivo [50]. It is also important to consider that the expression of HMGCS2 as well as of AKR1C3 was largely heterogeneous among the different cell lines. This heterogeneity might partly explain differences in treatment responses among the cell lines.

**Fig. 7:**
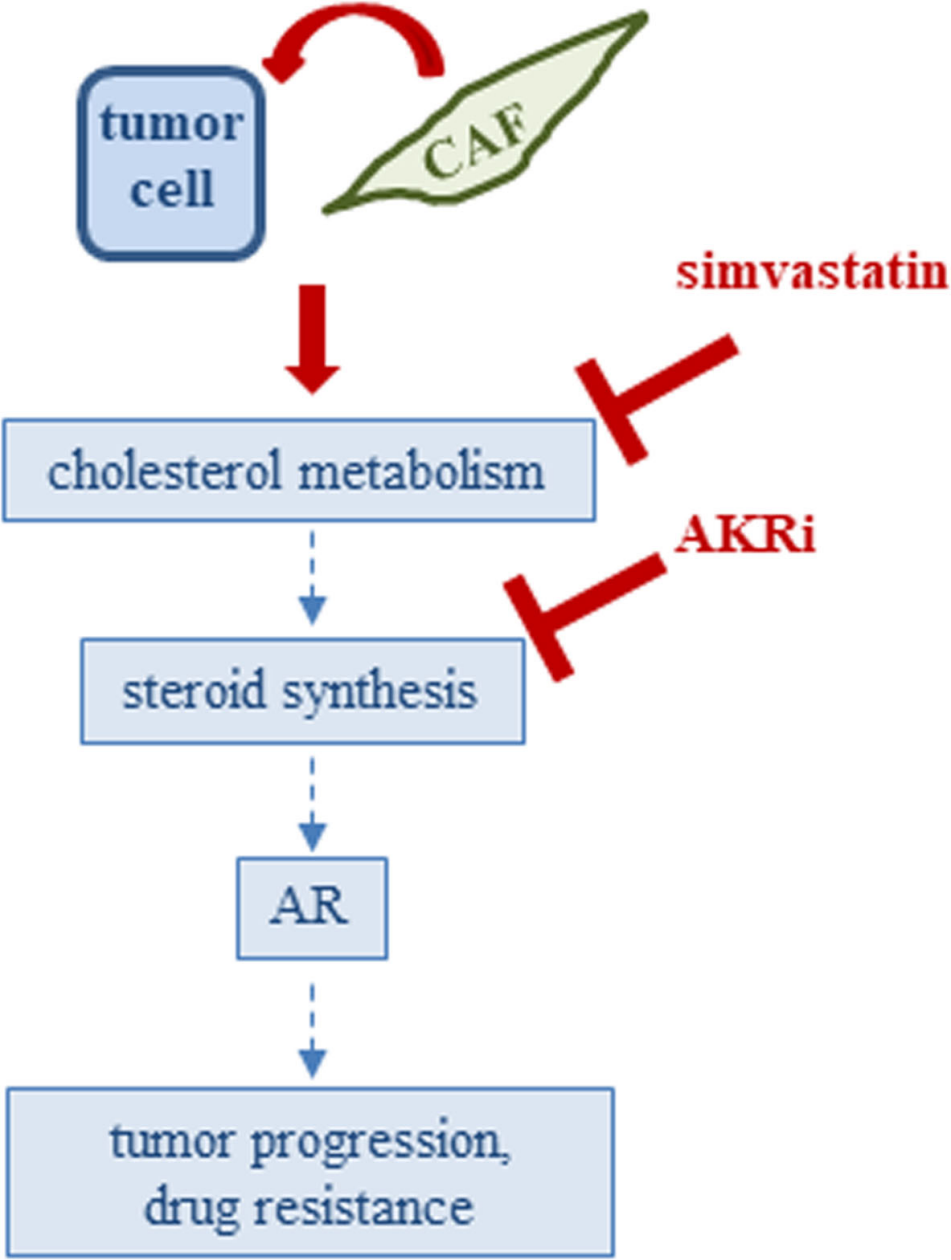
Schematic diagram showing combined targeting of cholesterol and steroid biosynthesis with simvastatin and AKRi in PCa co-culture spheroids

Based on this heterogeneity of the cells and the fact that cholesterol and steroid metabolism were increased in PCa cells upon co-culture with CAFs, rendering the cells less susceptible to the antiandrogen enzalutamide, we considered to simultaneously inhibit both pathways (Fig. 7). Importantly, combining simvastatin with the AKR1C3 inhibitor potentiated the growth-inhibitory effects of single drugs and effectively inhibited cell and spheroid growth of castration and enzalutamide resistant PCa cells.

## Conclusions

In summary, our data strongly suggest that CAFs induce an upregulation of cholesterol metabolism and steroid biosynthesis in PCa cells, enabling the tumor cells to escape AR targeted therapies. Though the mechanisms underlying this interaction between the tumor cells and the microenvironment still warrant further investigations, we could show that targeting cholesterol metabolism together with blockade of AKR1C3 to target steroid biosynthesis represents a promising way to inhibit castration and enzalutamide resistant PCa.

## Supplementary information

**Additional file 1.** Array data 3D vs 2D culture.

**Additional file 2.** Array data mono vs co-cultures.

## Data Availability

The datasets used and/or analyzed during the current study are available from the corresponding author on reasonable request.

## Abbreviations

3D: 3-dimensional
AKR1C3: Aldo-keto reductase type C3
AR: Androgen receptor
CAF: Cancer-associated fibroblasts
CRPC: Castration resistant prostate cancer
DMSO: Dimethyl sulfoxide
ECM: Extracellular matrix
GAPDH: Glyceraldehyde-3-phosphate dehydrogenase
HMBS: Hydroxymethylbilane synthase
HMGCS2: Hydroxymethyl glutaryl CoA synthase 2
IHC: Immunohistochemistry
PCa: Prostate cancer
PSA: Prostate specific antigen
